# Familial glucocorticoid deficiency presenting with hyperpigmentation, gigantism, and motor development delay: a case report

**DOI:** 10.1186/s13256-019-2206-5

**Published:** 2019-09-04

**Authors:** Kanchana Uyangoda, Phirarthana Kamalanathan, Sachith Mettananda

**Affiliations:** 1 Colombo North Teaching Hospital, Ragama, 11010 Sri Lanka; 2 Department of Paediatrics, Faculty of Medicine, University of Kelaniya, Thalagolla Raod, Ragama, 11010 Sri Lanka

**Keywords:** Adrenal disorders, Pediatric endocrinology, Pediatric neurology

## Abstract

**Background:**

Familial glucocorticoid deficiency is a rare autosomal recessive disorder characterized by isolated glucocorticoid deficiency. Most patients are diagnosed following episodes of hypoglycemia or convulsion. We report the case of an infant with familial glucocorticoid deficiency who presented with hyperpigmentation, gigantism, and motor developmental delay without documented hypoglycemia, convulsion, or circulatory collapse.

**Case presentation:**

A 10-month-old Sri Lankan Sinhalese baby boy born to consanguineous parents presented with generalized hyperpigmentation and overgrowth since birth. He had marginal gross motor developmental delay. His weight, length, and head circumference were above normal range for his age. Investigations revealed low serum cortisol and high adrenocorticotrophic hormone levels with no cortisol response following adrenocorticotropin stimulation. Serum electrolytes and aldosterone levels were normal. A diagnosis of familial glucocorticoid deficiency was made based on isolated glucocorticoid deficiency, hyperpigmentation, and tall stature.

**Conclusions:**

This case report highlights that glucocorticoid deficiency can present without documented hypoglycemia and circulatory collapse and a high degree of suspicion is needed in diagnosis.

## Background

Familial glucocorticoid deficiency (FGD) is a rare autosomal recessive disorder characterized by isolated glucocorticoid deficiency with normal mineralocorticoid activity [1]. Two main types are described: type 1, which is caused by mutations in the gene encoding for adrenocorticotrophic hormone (ACTH) receptor (melanocortin-2 receptor, *MC2R*); and type 2, due to a mutation in melanocortin-2 receptor associated protein (*MRAP*) [2, 3]. Most of the reported cases of patients with FGD were diagnosed following episodes of hypoglycemia, recurrent convulsions, or circulatory collapse. Here, we report the case of an infant with FGD who presented with hyperpigmentation, gigantism, and isolated gross motor developmental delay.

## Case presentation

**Fig. 1:**
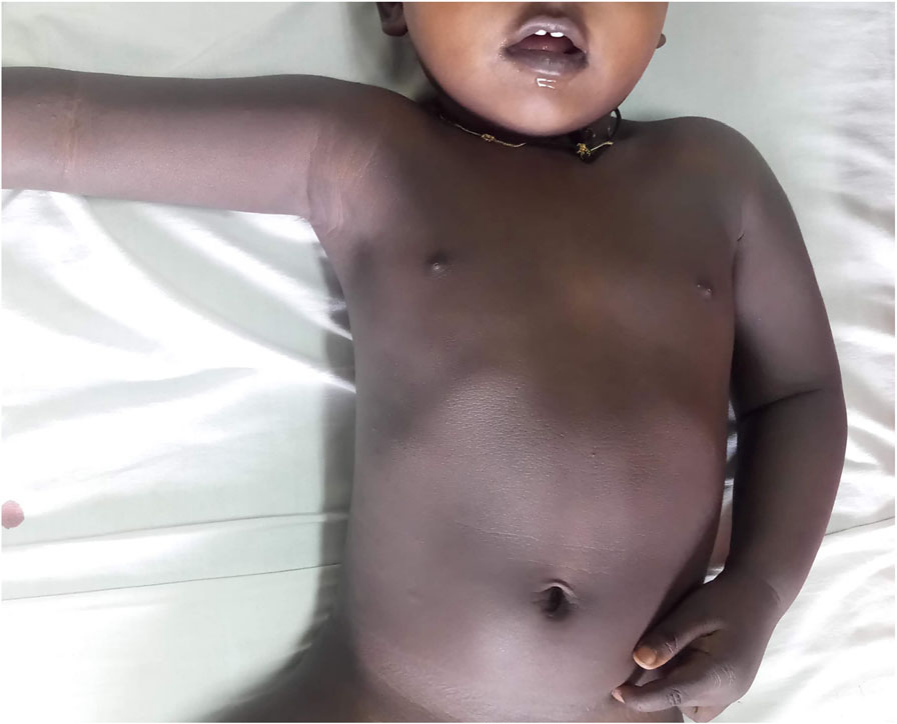
Photograph of the child demonstrating marked hyperpigmentation

**Fig. 2:**
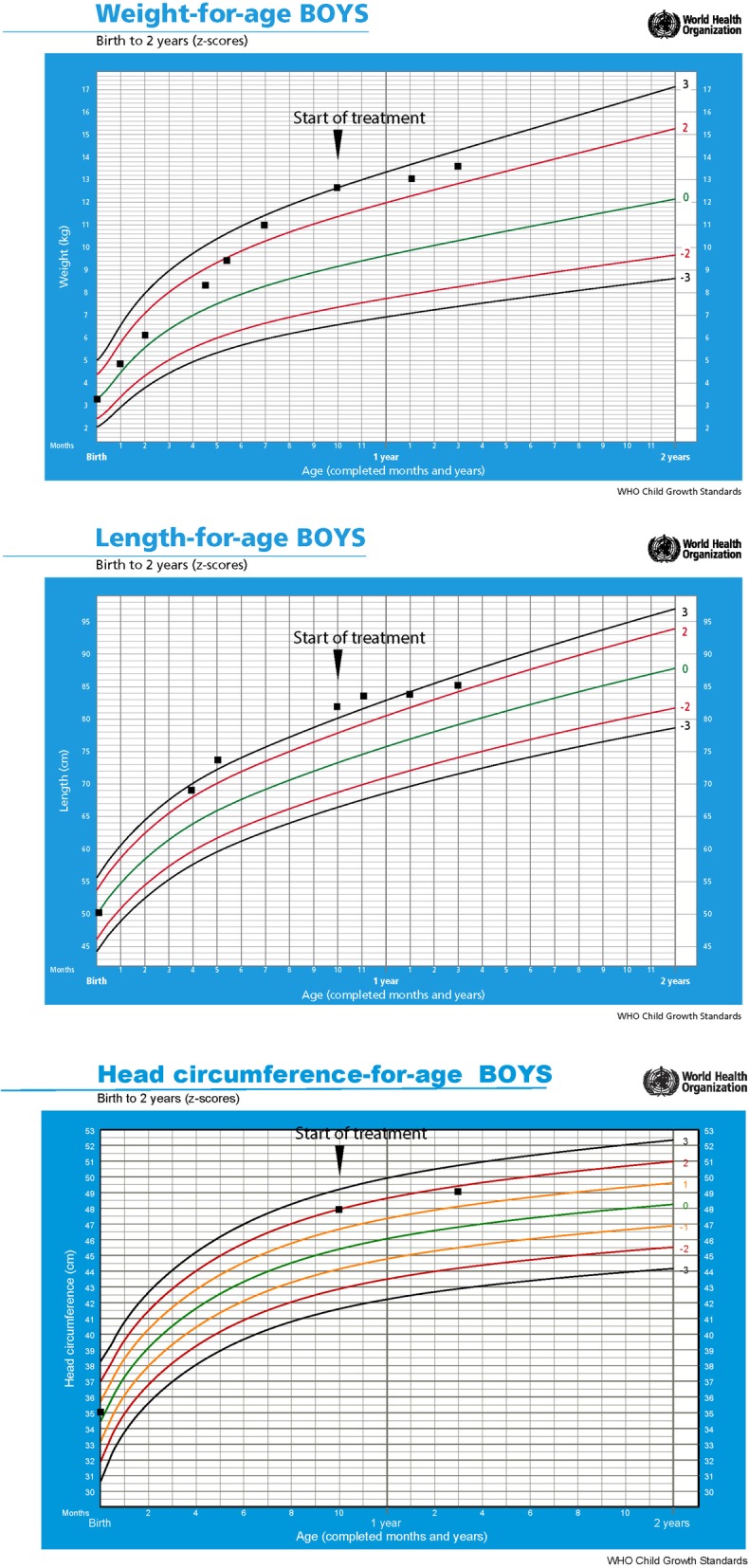
Growth charts demonstrating accelerated growth during first 10 months and slowing down of growth rate after commencement of treatment

A 10-month-old Sri Lankan Sinhalese baby boy presented with generalized hyperpigmentation and overgrowth since birth (Fig. 1). He was born at term following an uncomplicated antenatal period and had a normal perinatal period without episodes of hypoglycemia or circulatory collapse. He was the only child of a pair of consanguineous parents. His growth parameters at birth were within normal limits; weight 3.3 kg (at median for age), length 50 cm (at median for age), and head circumference 35 cm (between median and +1 SD). Since birth his growth chart demonstrated accelerated growth in weight, length, and head circumference (Fig. 2). His parents observed hyperpigmentation at birth which worsened gradually over time. His developmental history revealed marginal gross motor developmental delay; at 10 months of age, he was able to sit without support, however, he was unable to come to seated position on his own, stand with support, or crawl. His vision, fine motor, speech, and social development milestones were age appropriate. There were no previous hospital admissions, recurrent infections, seizures, episodes of shock, or documented hypoglycemia.

On examination, his length was 84 cm (above +3 SD), weight was 12.6 kg (at +3 SD), and head circumference was 48 cm (at +2 SD). He had generalized hyperpigmentation involving oral mucosa, palms, and soles. The rest of the physical examination, including cardiovascular system, blood pressure, abdomen, and genitalia was clinically normal.

Basic hematological and biochemical investigations that included full blood count, C-reactive protein, renal and liver function tests, and serum electrolytes were normal. His random blood glucose was 76 mg/dl. Subsequent investigations revealed very low serum cortisol (< 22 nmol/l; normal 55–304 nmol/l) and very high ACTH (> 1734 pg/ml; normal 10–50 pg/ml) suggesting glucocorticoid deficiency. His serum aldosterone level was normal. An ACTH stimulation test demonstrated markedly reduced basal cortisol levels (< 4.39 nmol/L) with no cortisol response following administration of synthetic ACTH (cortisol remained < 4.39 nmol/L, 30 and 60 minutes after ACTH stimulation) suggesting severe glucocorticoid deficiency. MRI of his brain showed normal pituitary gland with marginal dilatation of the trigone and the body of the lateral ventricles; however, there was no hydrocephalus or structural brain abnormalities.

FGD was diagnosed based on isolated glucocorticoid deficiency, hyperpigmentation, and tall stature. Molecular testing was not done due to unavailability. The baby was started on orally administered hydrocortisone 10 mg/m^2^ per day and his parents were counseled on the requirement for lifelong steroids. A review of this baby 5 months after initiating treatment revealed less pigmentation and slowing of growth in weight, length, and head circumference (Fig. 2) confirming the diagnosis.

## Discussion and conclusions

FGD is a rare disorder which is characterized by isolated glucocorticoid deficiency and tall stature presenting in the first decade of life [3, 4]. In most of the previous reports, patients presented with recurrent episodes of hypoglycemia with or without repeated convulsions. However, our patient did not have episodes of symptomatic hypoglycemia. Diagnosis of this child was based on hyperpigmentation and tall stature which led us to investigate for possible adrenal insufficiency.

Tall stature in FGD has been attributed to extremely high levels of ACTH acting through other melanocortin receptors, that is, melanocortin-1 receptor (MC1R) to melanocortin-5 receptor (MC5R), to affect bone and cartilage growth independent of growth hormone activity [3]. Five members of the melanocortin receptor family (MC1R to MC5R) are expressed in varying degrees in bone and high levels of ACTH acting on these receptors give rise to tall stature [5]. So far, to the best of our knowledge, all patients described in the medical literature have had tall stature which has not been associated with increased weight gain. In contrast, our patient had symmetrical overgrowth in all growth parameters including weight, height, and head circumference. Although FGD is not directly linked to increased weight gain, mutations in the related accessory protein melanocortin receptor accessory protein 2 (MRAP2), which is predominantly expressed in the hypothalamus, is associated with mammalian obesity [6]. It may be possible that weight gain in our patient is mediated through this protein. Excessive head growth is possibly mediated through the action of ACTH on bone growth through melanocortin receptors.

Another unusual finding of this case report is the isolated gross motor developmental delay. Although this is not previously reported in association with FGD, marginal isolated gross motor delay might have resulted from episodes of asymptomatic hypoglycemia which were not documented during the neonatal period. Also, excessive body weight could have contributed to marginal delay in gross motor milestones.

In conclusion, this case report highlights that glucocorticoid deficiency can present without documented hypoglycemia and circulatory collapse; therefore, a high degree of suspicion is needed in diagnosis. FGD should be suspected in children who present with hyperpigmentation with overgrowth and must be appropriately referred for early diagnosis and treatment to minimize long-term complications.

## Data Availability

Not applicable.

## Abbreviations

ACTH: Adrenocorticotrophic hormone
FGD: Familial glucocorticoid deficiency
MC1R: Melanocortin-1 receptor
MC2R: Melanocortin-2 receptor
MC5R: Melanocortin-5 receptor
MRAP: Melanocortin-2 receptor associated protein
MRAP2: Melanocortin receptor accessory protein 2
